# ALK inhibitor resistance in ALK^F1174L^-driven neuroblastoma is associated with AXL activation and induction of EMT

**DOI:** 10.1038/onc.2015.434

**Published:** 2015-11-30

**Authors:** D N Debruyne, N Bhatnagar, B Sharma, W Luther, N F Moore, N-K Cheung, N S Gray, R E George

**Affiliations:** 1 Department of Pediatric Hematology/Oncology, Dana-Farber Cancer Institute and Boston Children's Hospital, Boston, MA, USA; 2 Department of Pediatric Oncology, Memorial Sloan-Kettering Cancer Center, New York, NY, USA; 3 Department of Cancer Biology, Dana-Farber Cancer Institute, Boston, MA, USA; 4 Department of Biological Chemistry and Molecular Pharmacology, Harvard Medical School, Boston, MA, USA; 5 Department of Pediatrics, Harvard Medical School, Boston, MA, USA

## Abstract

The crizotinib-resistant *ALK*^*F1174L*^ mutation arises *de novo* in neuroblastoma (NB) and is acquired in *ALK* translocation-driven cancers, lending impetus to the development of novel anaplastic lymphoma kinase (ALK) inhibitors with different modes of action. The diaminopyrimidine TAE684 and its derivative ceritinib (LDK378), which are structurally distinct from crizotinib, are active against NB cells expressing *ALK*^*F1174L*^. Here we demonstrate acquired resistance to TAE684 and LDK378 in ALK^F1174L^-driven human NB cells that is linked to overexpression and activation of the AXL tyrosine kinase and epithelial-to-mesenchymal transition (EMT). AXL phosphorylation conferred TAE684 resistance to NB cells through upregulated extracellular signal-regulated kinase (ERK) signaling. Inhibition of AXL partly rescued TAE684 resistance, resensitizing these cells to this compound. AXL activation in resistant cells was mediated through increased expression of the active form of its ligand, GAS6, that also served to stabilize the AXL protein. Although ectopic expression of *AXL* and *TWIST2* individually in TAE684-sensitive parental cells led to the elevated expression of mesenchymal markers and invasive capacity, only *AXL* overexpression induced resistance to TAE684 as well. TAE684-resistant cells showed greater sensitivity to HSP90 inhibition than did their parental counterparts, with downregulation of AXL and AXL-mediated ERK signaling. Our studies indicate that aberrant AXL signaling and development of an EMT phenotype underlie resistance of ALK^F1174L^-driven NB cells to TAE684 and its derivatives. We suggest that the combination of ALK and AXL or HSP90 inhibitors be considered to delay the emergence of such resistance.

## Introduction

The predictable emergence of resistance to tyrosine kinase inhibitors (TKIs), leading to disease progression or relapse, has hindered their long-term therapeutic impact.^1^ This obstacle is best exemplified by the development of resistance to imatinib in *BCR-ABL*-expressing chronic myeloid leukemia and gefitinib in *EGFR*-mutant non-small-cell lung cancer,^2, 3^ and is likely to impede efforts to devise effective targeted therapy for many other cancers, including neuroblastoma (NB). This aggressive childhood tumor is characterized by mutations in the anaplastic lymphoma kinase (ALK) receptor tyrosine kinase (RTK) in 10% of cases.^4, 5, 6, 7^ Many of these point mutations are considered ‘drivers' of the malignant process: not only do they induce constitutive, ligand-independent activation of ALK signaling, but also their inhibition leads to tumor cell death and tumor regression.^5, 6^ The most common somatic mutation in NB, *ALK*^*F1174L*^, is highly tumorigenic, both by itself and when coexpressed with the *MYCN* oncogene, a combination that increases the penetrance of the disease and accelerates tumor formation.^8, 9^ This mutation confers primary resistance to the ALK inhibitor crizotinib in NB^9^ and serves as a mechanism of acquired resistance to crizotinib in patients with *ALK*-translocated cancers.^10^

Several structurally unrelated small molecule ALK inhibitors have been developed such as alectinib (CH5424802) that has shown activity against *ALK*^*F1174L*^-positive tumors.^11^ Similarly, the lead compound TAE684,^12^ from which the recently Food and Drug Administration (FDA)-approved inhibitor ceritinib (LDK378, Novartis) is derived,^13^ has exhibited potent activity against ALK^F1174L^ in both *ALK* translocation-positive cancers^10^ and NB.^5, 14^ Nonetheless, resistance to these ATP-competitive agents will inevitably develop as a consequence of their wider clinical application. We therefore sought to elucidate the mechanism(s) underlying acquired resistance to ALK inhibitors in ALK^F1174L^-driven NB as a means to uncover secondary targets that could be exploited to prolong responses in these patients. By generating TAE684 and LDK378 resistance models of *ALK*^*F1174L*^-positive human NB cells, we identified overexpression and GAS6-mediated activation of a TAM family RTK, AXL, as the principal resistance-related alteration in these cells. This change was associated with activation of the mitogen-activated protein kinase (MAPK) signaling pathway and the development of an epithelial-to-mesenchymal transition (EMT) phenotype. Importantly, inhibition of AXL with a small molecule inhibitor led to decreased growth and invasiveness of the resistant cells with a concomitant decrease in extracellular signal-regulated kinase (ERK) signaling. We also demonstrate that HSP90 inhibition, through its impact on AXL binding, induces striking cytotoxicity in TAE684-resistant cells. Hence, we suggest that the combination of ALK and AXL or HSP90 inhibition could serve as part of an effective strategy of targeted therapeutics for ALK^F1174L^-driven NB and other tumors dependent on this aberrant RTK.

## Results

### TAE684 resistance is associated with the loss of ALK activity but maintenance of downstream signaling

**Figure 1:**
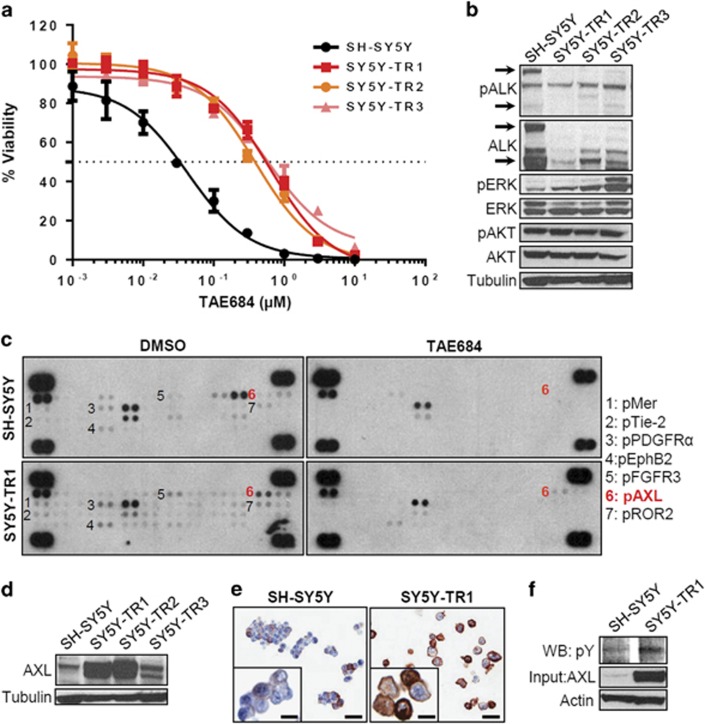
Development of TAE684 resistance is associated with activation of AXL in *ALK*-mutated SH-SY5Y NB cells. (**a**) Dose–response curves for parental SH-SY5Y cells and three TAE684-resistant clones (SY5Y-TR1 to TR3) treated with increasing concentrations of TAE684 for 72 h (half-maximal inhibitory concentration (IC_50_)=SH-SY5Y, 31 nM; SY5Y-TR1, 521 nM; SY5Y-TR2, 374 nM; SY5Y-TR3, 581 nM). (**b**) Western blot analysis of total and pALK and indicated downstream signaling molecules in TAE684-sensitive and -resistant SH-SY5Y cells. Arrows indicate ALK products of 220 and 140 kDa. (**c**) Phospho-proteomic analysis of RTKs in SH-SY5Y and SY5Y-TR1 cells exposed to either dimethyl sulfoxide (DMSO) or TAE684 (1 μM) for 6 h. Each RTK is shown in duplicate, and the pairs in each corner of the array are positive controls. Significantly regulated RTKs are numbered. (**d**) Western blot analysis of total AXL expression in parental SH-SY5Y and the three SY5Y-TR clones. (**e**) Immunocytochemical analysis of AXL expression in SH-SY5Y and SY5Y-TR1 cells (scale bars, 10 μm, insets, 5 μm). (**f**) Analysis of pAXL in SH-SY5Y and SY5Y-TR1 cells in which AXL was immunoprecipitated using an anti-AXL antibody, followed by western blotting with an anti-phosphotyrosine antibody. Tubulin or actin were used as loading controls in all western blots.

NB cells that express the *ALK*^*F1174L*^ mutation are relatively resistant to crizotinib but are sensitive to TAE684.^5, 9^ To elucidate the mechanisms of resistance to ALK^F1174L^ inhibitors, we first established TAE684-resistant cells (SH-SY5Y-TR) through continuous exposure of SH-SY5Y cells to increasing doses of the compound over 8–12 months (Supplementary Figure S1a). Three individual subclones (SH-SY5Y-TR1, SH-SY5Y-TR2 and SH-SY5Y-TR3) were expanded (Figure 1a), and subsequently maintained in ∼35 times the half-maximal inhibitory concentration (IC_50_) of TAE684.

Resistance to targeted therapy can arise from either secondary mutations in the drug target itself or upregulation of compensatory pathways.^1^ We therefore first analyzed the phosphorylation status of ALK and its downstream targets in SH-SY5Y-TR-resistant cells. As compared with results in parental cells, ALK phosphorylation was decreased in both the primary resistant pool (Supplementary Figure S1b) and the three subclones (Figure 1b), suggesting that the acquired resistance was not mediated by secondary mutations in *ALK*. This result was confirmed by sequence analysis and genomic PCR showing the absence of mutations other than *F1174L* or gene amplification, respectively (data not shown). The absence of ALK phosphorylation also ruled out upregulation of drug efflux transporters such as the ABC (ATP-binding cassette) superfamily as a potential mechanism of resistance, as ALK would remain phosphorylated if this were to be the case.

The *ALK*^*F1174L*^ mutation activates the phosphatidylinositol-3-kinase/AKT/mammalian target of rapamycin (PI3K/AKT/mTOR) and MAPK/ERK pathways in NB cells, both of which are downregulated when ALK is inhibited.^5, 9^ Despite decreased levels of pALK, AKT activation was maintained in the resistant SH-SY5Y-TR pool as well as all three SH-SY5Y-TR subclones (Figure 1b and Supplementary Figure S1b). Importantly, compared with parental cells, ERK phosphorylation was increased in SH-SY5Y-TR cells and its subclones (Figure 1b and Supplementary Figure S1b). The upregulated ERK signaling in the context of suppressed ALK phosphorylation suggested the development of an alternative mechanism of resistance, most likely activation of another tyrosine kinase capable of bypassing TAE684 inhibition.

### The AXL receptor tyrosine kinase is upregulated in TAE684-resistant NB cells

To identify upstream RTKs that may contribute to TAE684 resistance, we compared the phosphorylation status of 42 candidates in SH-SY5Y and SH-SY5Y-TR1 cells before and after treatment with TAE684 (Figure 1c). Parental SH-SY5Y cells showed basal activation of several RTKs, most of which were decreased or lost upon TAE684 treatment (Figure 1c). Under dimethyl sulfoxide (DMSO) treatment conditions, TAE684-resistant SH-SY5Y-TR1 cells showed enhanced phosphorylation of seven additional RTKs (MER, TIE-2, PDGFRα, EPHB2, FGFR3, AXL and ROR2). Two of these, MER and AXL, belonged to the same TAM receptor tyrosine kinase family whose aberrantly elevated signaling has been linked to cancer progression, metastasis and resistance to therapy.^15^ Acute exposure to TAE684 led to complete loss of phosphorylation of all of these candidates except for AXL and EPHB2. Thus, the sustained upregulation of AXL and EPHB2 in the SH-SY5Y-TR1-resistant cells suggested a role for these RTKs in mediating resistance to TAE684.

We selected AXL for further study because of the known role of this transmembrane receptor in mediating drug resistance, especially to TKIs.^16, 17^ We confirmed that AXL expression was markedly increased in two of the three resistant clones and marginally in the third (Figure 1d). Intense membrane staining of AXL was apparent on immunocytochemical staining of SH-SY5Y-TR1 cells (Figure 1e). Interestingly, parental SH-SY5Y cells also contained a minor subpopulation of *AXL*-expressing cells compared with TAE684-resistant cells (18 of 100 cells vs 94 of 100 resistant cells), suggesting the pre-existence and clonal selection of cells expressing AXL under drug pressure to compensate for the loss of ALK signaling. Finally, the increased protein expression was accompanied by phosphorylation of AXL in SH-SY5Y-TR1 cells (Figure 1f), suggesting activation of the tyrosine kinase. Further evidence to support AXL activation as a key mediator of TAE684 resistance came from experiments with an additional ALK^F1174L^-driven NB cell line, SK-N-SH. SK-N-SH cells rendered resistant to TAE684 (SK-N-SH-TAE-R) showed upregulation of AXL as well as ERK signaling despite downregulation of phosphorylated ALK (Supplementary Figures S2a and b). Together, these results indicate that TAE684-resistant NB cells express higher levels of alternative RTKs such as AXL to compensate for the loss of ALK phosphorylation.

### AXL activation confers resistance to TAE684 in ALK^F1174L^-driven NB cells

**Figure 2:**
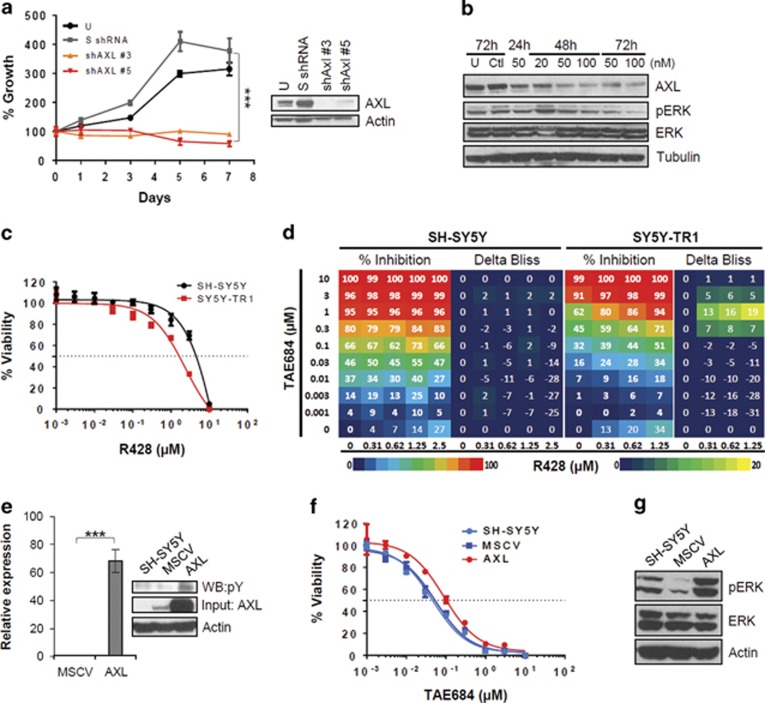
TAE684-resistant cells are dependent on AXL signaling. (**a**) Growth curve (left) of untransfected (U), scrambled (S shRNA) or *AXL* shRNA-expressing SY5Y-TR1 cells. Two different *AXL* shRNAs (3 and 5) were used. The results represent the mean±s.d. of three separate experiments. ***P<0.001. Western blot analysis (right) of AXL knockdown in the same cells. (**b**) Western blot analysis of AXL and pERK in SY5Y-TR1 cells expressing either a nonspecific (Ctl) or AXL siRNA at the indicated doses for the indicated times. Untransfected SY5Y-TR1 cells (U) were also used as controls. (**c**) Dose–response curves for SH-SY5Y and SY5Y-TR1 cells treated with the AXL small molecule inhibitor, R428, for 3 days (IC_50_=SH-SY5Y, 4.79 μM; SY5Y-TR1, 1.93 μM). (**d**) Drug matrix illustrating percent inhibition (left) and Delta Bliss (right) for R428 in combination with TAE684 in SH-SY5Y and SY5Y-TR1 cells. Percent inhibition values were derived from the cell viability assay after 3 days of treatment with the two compounds. Considering *A* and *B* to be the fractional growth inhibition (percent inhibition divided by 100) of single drug treatment at a given dose (for example, TAE684 and R428, respectively), a Bliss expectation was calculated using the formula: (*A*+*B*)−*A* × *B* for every drug concentration used. The ‘Delta Bliss' represents the difference between the Bliss expectation and the observed fractional growth inhibition of the combination of drug *A* and *B* at the same dose. A Delta Bliss of 1 indicates an additive effect, whereas a value <0 or >1 indicate antagonistic and synergistic effects of the combination, respectively. Color scales for each specific grid (left=percent inhibition; right=Delta Bliss) are shown. Values represent the mean of two separate experiments. (**e**) qRT–PCR analysis (left) of AXL expression in untransfected (SH-SY5Y) or SH-SY5Y cells stably expressing either AXL (AXL) or vector control (MSCV). ***P<0.001. Western blot analysis (right) of total and pAXL in these cells. AXL was immunoprecipitated using an anti-AXL antibody and western blotting performed with an anti-phosphotyrosine antibody. (**f**) Dose–response curves for cells in (**e**) exposed to TAE684 for 3 days (IC_50_=SH-SY5Y, 44 nM; SH-SY5Y MSCV, 50 nM; SH-SY5Y AXL, 102 nM). (**g**) Western blot analysis of total and pERK in the same cells as in (**e**).

To clarify the role of AXL activation in resistance to TAE684, we first abrogated its expression in SH-SY5Y-TR1 cells using small hairpin RNA (shRNA) knockdown, noting a significant decrease in the growth of AXL-depleted compared with untransfected or control shRNA-expressing cells (Figure 2a). AXL depletion was associated with a decrease in pERK levels at 72 h compared with untransfected or control shRNA-expressing cells at the same time point (Figure 2b). SH-SY5Y-TR1 cells were three times more sensitive to the AXL inhibitor R428^(ref. 18)^ than were parental SH-SY5Y cells (Figure 2c). Similar results were seen in the SK-N-SH-TAE-R cells (Supplementary Figure S2c). Importantly, R428 also restored sensitivity to TAE684 in SH-SY5Y-TR1 cells, with a combination of the two agents having an additive effect (Figure 2d). Similar effects were not seen in TAE684-sensitive parental SH-SY5Y cells, possibly reflecting the dependency of the resistant cells on the proliferative and migratory effects of activated AXL. Next, we determined whether overexpression of AXL was sufficient to confer resistance to ALK inhibitors in *ALK*-mutated NB cells. Ectopic expression of *AXL* in TAE684-sensitive SH-SY5Y cells resulted in a twofold decrease in sensitivity to TAE684 (Figures 2e and f). Overexpression of *AXL* also led to an increase in pERK in these cells (Figure 2g). These findings suggest that AXL overexpression contributes to TAE684 resistance in ALK^F1174L^-driven NB cells and underscores the potential of AXL inhibition as a means to sensitize these resistant cells.

### Resistance to the ALK inhibitor LDK378 is also associated with AXL activation

**Figure 3:**
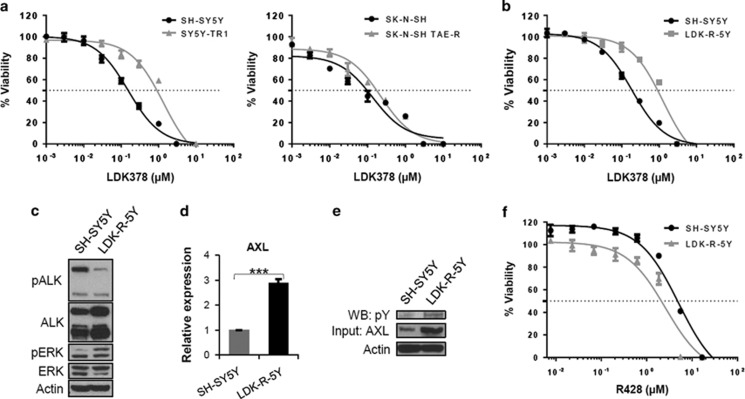
Resistance to LDK378 in ALK^F1174L^-driven SH-SY5Y cells is also associated with AXL upregulation. (**a**) Dose–response curves for parental SH-SY5Y and TAE684-resistant SY5Y-TR1 cells (left) treated with LDK378 for 3 days (IC_50_=SH-SY5Y, 150 nM; SY5Y-TR1, 1101 nM), and parental SK-N-SH and TAE684-resistant SK-N-SH-TAE-R cells (right) treated with LDK378 for 3 days (IC_50_=SK-N-SH, 69.4 nM; SK-N-SH TAE, 149.7 nM). (**b**) Dose–response curves for parental and LDK378-resistant SH-SY5Y (LDK-R-5Y) cells treated with LDK378 for 3 days (IC_50_=SH-SY5Y, 184 nM; LDK Res 5Y, 1049 nM). (**c**) Western blot analysis of total and pALK and ERK in parental and LDK-R-5Y cells. (**d**) qRT–PCR analysis of AXL in SH-SY5Y and LDK-R-5Y cells. ***P<0.001. (**e**) Analysis of pAXL in parental and LDK-R-5Y cells in which AXL was immunoprecipitated using an anti-AXL antibody, followed by western blotting with an anti-phosphotyrosine antibody. (**f**) Dose–response curves for parental and LDK-R-5Y cells treated with R428 for 3 days (R428: IC_50_=SH-SY5Y, 4420 nM; LDK-R-5Y, 2260 nM).

To extend findings with TAE684 to the more clinically relevant ALK inhibitor LDK378 (ceritinib), we first evaluated the sensitivity of both TAE684-resistant SH-SY5Y-TR1 and SK-N-SH-TAE-R cells to LDK378. These experiments showed cross-resistance between the two compounds (Figure 3a), suggesting that AXL activation might serve as a mechanism of resistance to newer ALK inhibitors. To support this hypothesis further, we generated LDK378-resistant SH-SY5Y cells (designated LDK-R-5Y), following the same procedure as used with TAE684 (Figure 3b). Similar to the findings in TAE684-resistant cells, pALK was downregulated in the LDK-R-5Y cells whereas pERK was upregulated (Figure 3c). Moreover, increased expression of AXL at both the mRNA and protein levels, as well as increased phosphorylation of AXL, were observed (Figures 3c–e). LDK-R-5Y cells were more sensitive to AXL inhibition by R428 than were the parental cells (Figure 3f). Thus, activation of AXL and ERK signaling appears to underlie acquired resistance of ALK^F1174L^-driven NB cells to both TAE684 and LDK378.

### TAE684-resistant SH-SY5Y-TR1 cells display functional EMT features

**Figure 4:**
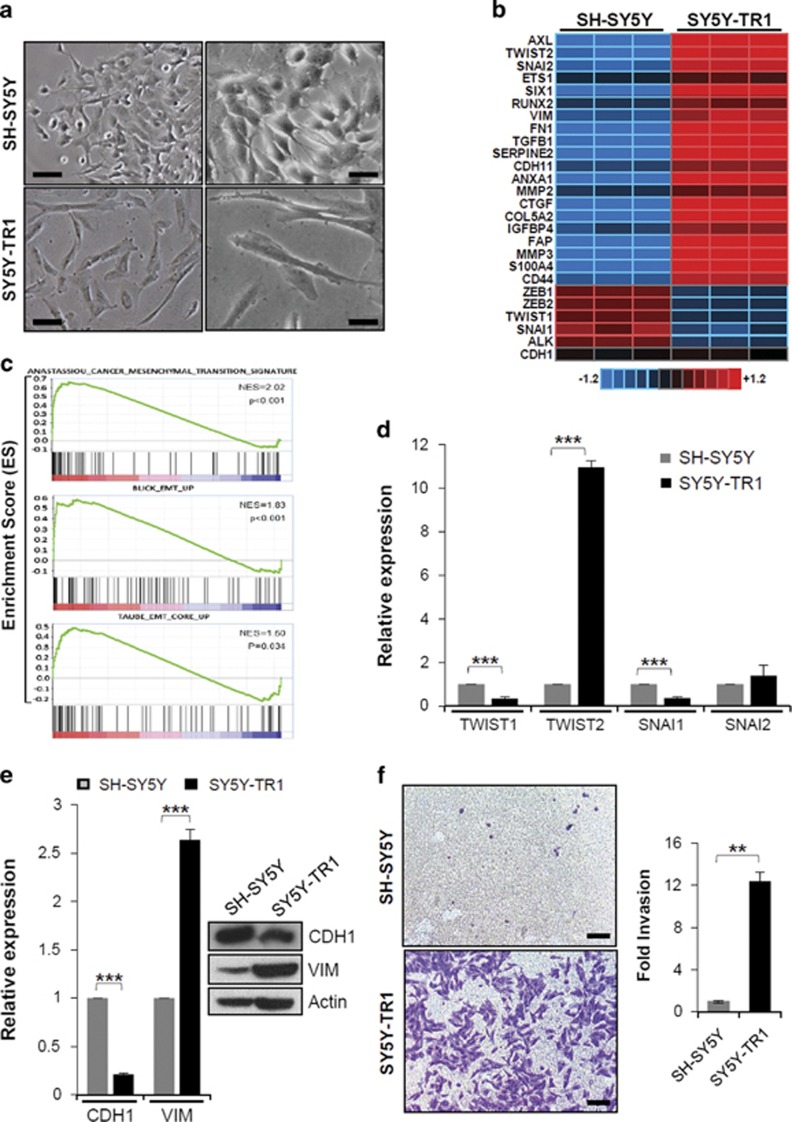
TAE684-resistant SH-SY5Y cells exhibit features of EMT. (**a**) Phase contrast micrographs of SH-SY5Y and SY5Y-TR1 cells (scale bars, 50 μm, inserts, 25 μm). (**b**) Representative heatmap showing differential expression of key EMT-related genes, as well as ALK and AXL, in TAE684-sensitive SH-SY5Y and -resistant SY5Y-TR1 cells. Fold change ⩾2.0; corrected *P*<0.05). (**c**) GSEA of three previously published EMT gene signatures in SY5Y-TR1 cells as compared with SH-SY5Y cells. NES, normalized enrichment score. (**d**) qRT–PCR analysis of the indicated genes in SH-SY5Y and SY5Y-TR1 cells. ***P<0.001. (**e**) mRNA and protein levels of E-cadherin (CDH1) and vimentin (VIM) in SH-SY5Y and SY5Y-TR1 cells, as analyzed by qRT–PCR (left) and western blotting (right). ***P<0.001. (**f**) Invasion potential (left) of SH-SY5Y and SY5Y-TR1 cells as analyzed by matrigel assay (scale bar, 50 μm). Quantification of invasion potential (right) of the same cells. **P<0.01.

We noted that SH-SY5Y-TR1 cells exhibited striking morphological differences when compared with parental SH-SY5Y cells (Figure 4a). Whereas the parental cells were smaller in size with epithelioid morphology, SH-SY5Y-TR1 cells were elongated, spindle shaped and fibroblast-like, with decreased cell-to-cell contact, consistent with EMT. To determine whether these morphological alterations had an underlying molecular basis, we compared the gene expression profiles of the SH-SY5Y-TR1 cells with those of parental cells (Figure 4b). In addition to upregulation of *AXL* itself, the resistant cells showed significant differential expression of genes with major roles in EMT, with overexpression of the key transcriptional inducers *TWIST2* and *SNAI2*, and the characteristic mesenchymal markers vimentin (*VIM*) and fibronectin (*FN1*) (Figure 4b). Gene set enrichment analysis (GSEA) of the differentially expressed genes indicated significant enrichment of three distinct EMT-related gene signatures (Figure 4c).^19, 20, 21^ Overexpression of *TWIST2* in the TAE684-resistant cells was confirmed by quantitative reverse transcriptase–PCR (qRT–PCR; Figure 4d). Although E-cadherin (*CDH1*), a key marker of the epithelial cell state, was not significantly altered in the expression signatures (Figure 4b), quantitative PCR analysis demonstrated decreased mRNA levels of this gene as well as increased expression of vimentin in the resistant compared with the parental cells, findings that were also reflected at the protein level (Figure 4e). SH-SY5Y-TR1 cells also displayed significantly increased invasive properties when compared with parental cells on matrigel assays, attesting to their metastatic potential (Figure 4f). Together, these results suggest that acquired resistance to TAE684 by *ALK*-mutated NB cells is associated with functional EMT.

### AXL activation leads to EMT features and resistance to TAE684 through different mechanisms

**Figure 5:**
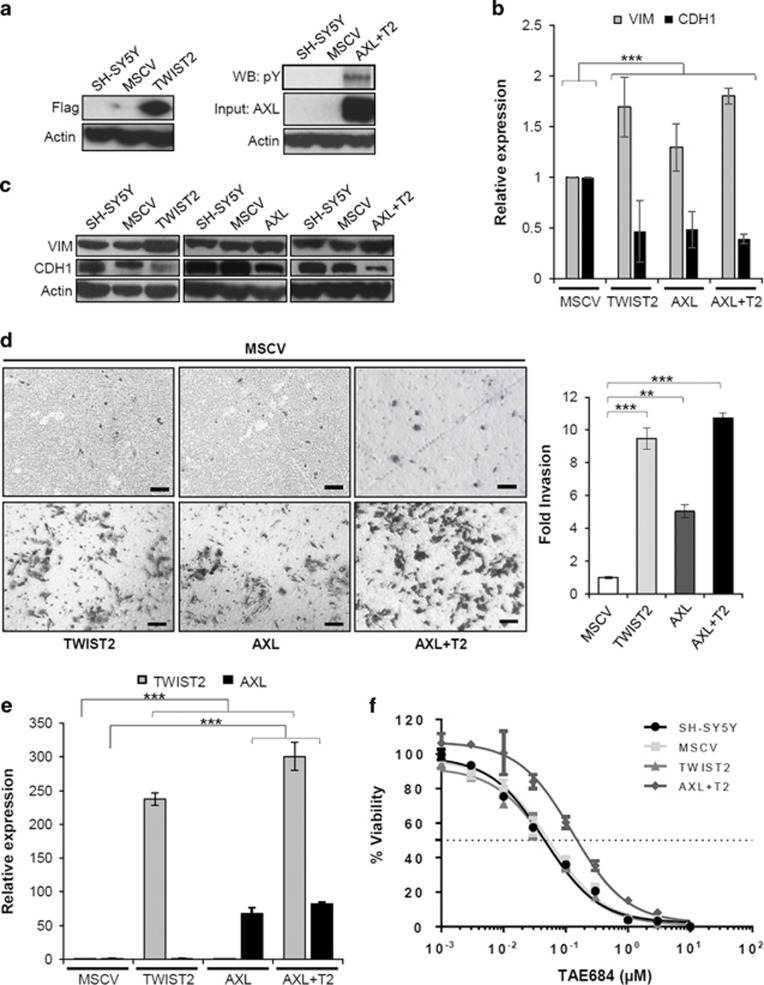
AXL overexpression in SH-SY5Y cells is sufficient to confer both mesenchymal features and TAE684 resistance. (**a**) Western blot analysis (left) of TWIST2 expression in SH-SY5Y cells overexpressing either a vector control (MSCV) or TWIST2 (TWIST2). Western blot analysis (right) of AXL expression and phosphorylation in cells engineered to express both AXL and TWIST2 together (AXL+T2). AXL was immunoprecipitated with an anti-AXL antibody, followed by western blotting with an anti-phosphotyrosine antibody. (**b**) qRT–PCR analysis of cadherin (CDH1) and vimentin (VIM) in SH-SY5Y cells expressing either TWIST2 or AXL singly or in combination. ***P<0.001. (**c**) Western blot analysis of CDH1 and VIM expression in the same cells as in (**a**). (**d**) Photomicrographs (left) showing invasion capacities, together with quantification (right) of the same cells as in (**b**) (scale bar, 50 μm). **P<0.01, ***P<0.001. (**e**) qRT–PCR analysis of expression levels of TWIST2 and AXL in the same cells. ***P<0.001. (**f**) Dose–response curves for the same cells as in (**a**) exposed to TAE684 for 3 days (IC_50_=SH-SY5Y, 44 nM; SH-SY5Y MSCV, 50 nM; SH-SY5Y TWIST2, 42 nM; SH-SY5Y AXL+T2, 155 nM).

Although AXL activation and EMT induction have both been linked to TKI resistance,^15, 22^ it is not clear whether EMT induction is an inevitable consequence of AXL activation, or vice versa. We therefore sought to induce EMT features in parental TAE684-sensitive cells and to determine whether this effect would cause increased expression of AXL or, indeed, altered sensitivity to TAE684. Overexpression of FLAG-tagged *TWIST2* (Figure 5a, left), the top EMT-associated gene that was differentially expressed in the resistant cells (Figure 4b), led to significantly elevated vimentin and decreased cadherin levels (Figures 5b and c) in parental SH-SY5Y cells as compared with vector-control transfected cells, as well as increased invasive capacity (Figure 5d). AXL expression, however, was not elevated in SH-SY5Y-TWIST2-expressing cells compared with control cells (Figure 5e). Moreover, these cells remained as sensitive to TAE684 as untransfected or vector control-expressing cells (Figure 5f). In contrast, as shown previously, ectopic expression of *AXL* in parental TAE684-sensitive SH-SY5Y cells caused a twofold reduction in sensitivity to TAE684 (Figures 2e and f), although it did not alter *TWIST2* expression levels (Figure 5e). Overexpression of *AXL* also led to a mesenchymal phenotype characterized by significant modulation of vimentin and cadherin expression (Figures 5b and c) and increased invasiveness (Figure 5d). Finally, simultaneous expression of both proteins in parental SH-SY5Y cells (Figure 5a, right) led to differential expression of vimentin and cadherin (Figures 5b and c), a highly invasive phenotype (Figure 5d) and, importantly, more than threefold decrease in sensitivity to TAE684 (Figure 5f). Together, these observations suggest that, in our model, combined expression of *AXL* and *TWIST2* leads to additive effects on EMT and drug resistance, whereas only AXL activation is associated with resistance to TAE684.

### AXL activation is facilitated by elevated levels of the cleaved form of its ligand GAS6

The dependence of TAE684 resistance in NB cells on AXL activation led us to investigate the mechanism of *AXL* upregulation in SH-SY5Y-TR1 cells. Gene amplification was excluded at the outset through quantitative PCR analysis of genomic DNA (Supplementary Figure S3a). Sequencing of the *AXL* promoter and coding regions likewise failed to identify mutations within SH-SY5Y-TR1 cells that could account for elevated AXL expression (data not shown). Methylation-specific PCR and bisulfite sequencing failed to reveal differential CpG hypomethylation as a mechanism of *AXL* overexpression, as previously reported^23^ (data not shown). We also assessed the contribution of miR-34a and miR-199a/b, the two acknowledged modulators of AXL expression,^24^ noting a decrease in miR-199b levels in the TAE684-resistant cells relative to the sensitive ones (Supplementary Figure S3b). Forced expression of miR-199b in SH-SY5Y-TR1 cells, however, failed to induce downregulation of *AXL* at either the mRNA (Supplementary Figure S3c) or protein (Supplementary Figure S3d) level. Notably, no effect on pERK levels (Supplementary Figure S3d) was seen, suggesting that aberrant microRNA regulation was not a major determinant of the increased *AXL* expression seen in the TAE684-resistant NB cells.

**Figure 6:**
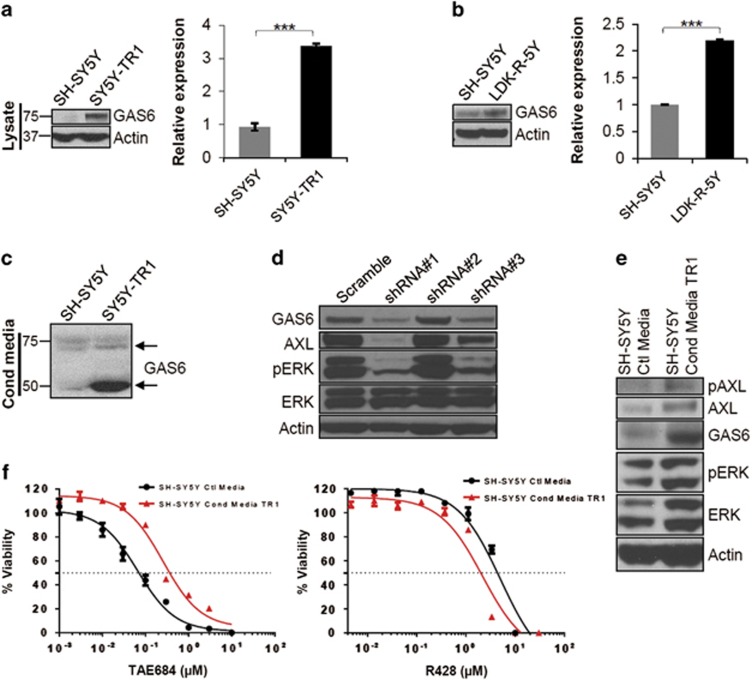
The AXL ligand, GAS6, is highly expressed in TAE684- and LDK378-resistant cells and leads to AXL activation. (**a**) Western blot analysis of GAS6 levels in cell lysates from parental SH-SY5Y and TAE684-resistant SY5Y-TR1 cells (left). qRT–PCR analysis of the GAS6-SV variant in the same cells (right). ***P<0.001. (**b**) Western blot (left) and qRT–PCR analysis (right) of the GAS6-SV variant in the parental and LDK378-resistant SH-SY5Y cells. ***P<0.001. (**c**) Western blot analysis of the 50 kDa cleaved GAS6 product in conditioned media from SH-SY5Y and SY5Y-TR1 cells. Arrows indicate both full-length and cleaved GAS6 forms. (**d**) Western blot analysis of the indicated proteins in SY5Y-TR1 cells expressing either GAS6 shRNAs or a control shRNA (scramble). Three different shRNAs (1, 2 and 3) were used. (**e**) Western blot analysis of total and pAXL and ERK levels, as well as GAS6 protein in SH-SY5Y cultivated for 3 days in conditioned media from control SH-SY5Y or TAE684-resistant SY5Y-TR1 cells. (**f**) Dose–response curves for parental SH-SY5Y cultivated for 3 days in conditioned (Cond) media from control SH-SY5Y or TAE684-resistant SY5Y-TR1 cells and then treated with TAE684 or R428 for 3 days (TAE684: IC_50_=SH-SY5Y Ctl Media, 66 nM; SH-SY5Y Cond Media TR1, 385 nM; R428: IC_50_=SH-SY5Y Ctl Media, 4085 nM; SH-SY5Y Cond Media TR1, 1836 nM).

AXL receptor dimerization and activation through autophosphorylation has been reported to occur through binding of its physiological ligand GAS6 that triggers a cascade of intracellular signaling events that culminate in cell proliferation and survival.^25^ *GAS6* is normally expressed in two forms: the full-length 75 kDa protein and a slightly larger protein, GAS6-SV (86 kDa), because of alternative splicing.^26^ This modification leads to the insertion of a 43-amino-acid sequence that contains a consensus cleavage site, whose proteolytic cleavage results in a soluble 50 kDa product that is ultimately responsible for AXL receptor activation.^26^ We observed a marked increase in GAS6 protein levels in TAE684-resistant SH-SY5Y-TR1 cells compared with parental cells, as well as significantly higher mRNA levels of the GAS6 splice variant, GAS6-SV (Figure 6a). Importantly, the same findings, in conjunction with *AXL* upregulation, were observed in SH-SY5Y cells that were made resistant to LDK378 (Figure 6b). Moreover, analysis of conditioned media from SH-SY5Y-TR1 cells revealed an abundance of the cleaved active 50 kDa GAS6 fragment (Figure 6c). Hence, the presence of increased levels of GAS6, as well as that of its active secreted form in TAE684-resistant cells, suggested that AXL activation in these cells resulted from upregulation of its ligand. To test this hypothesis, we depleted *GAS6* in SH-SY5Y-TR1 cells through shRNA knockdown, noting a resultant significant decrease in AXL levels and, more importantly, concomitant attenuation of ERK activation (Figure 6d). Moreover, conditioned media from SH-SY5Y-TR1 cells led to increased levels of GAS6 and activated AXL proteins, as well as upregulated ERK signaling in TAE684-sensitive parental SH-SY5Y cells (Figure 6e). These cells became less sensitive to TAE684 while showing increased sensitivity to R428 (Figure 6f). We also noted that *AXL* mRNA levels in both parental and *AXL*-overexpressing SH-SY5Y cells did not increase after treatment with human recombinant GAS6 (rGAS6), arguing against a positive feedback loop on AXL production (Supplementary Figure S4a). Rather, cells that were pretreated with rGAS6 showed higher AXL protein levels in the presence of the protein synthesis inhibitor cycloheximide, suggesting that GAS6 upregulation led to AXL protein stabilization (Supplementary Figure S4b). Together, these results suggest that AXL activation in SHSY5Y-TR1 cells is facilitated by increased production of soluble GAS6 via aberrant expression of cleavable GAS6-SV.

### TAE684-resistant AXL-activated SH-SY5Y cells are sensitive to HSP90 inhibition

**Figure 7:**
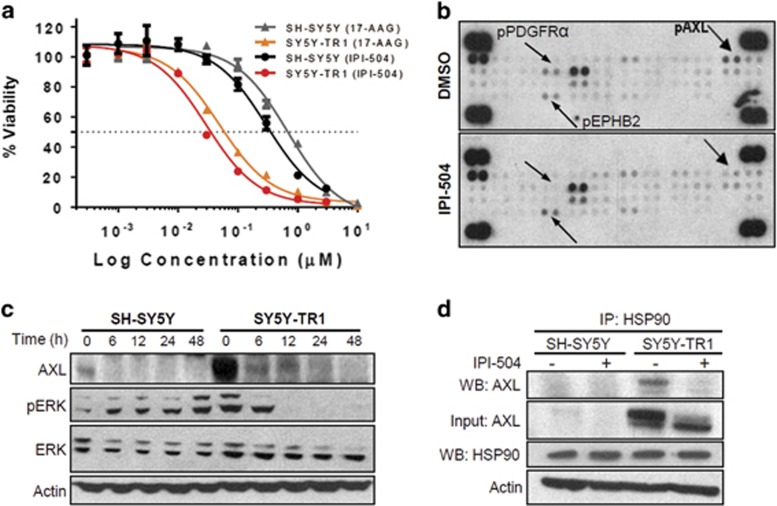
TAE684-resistant SY5Y-TR1 cells are sensitive to HSP90 inhibition. (**a**) Dose–response curves for parental SH-SY5Y and TAE684-resistant SY5Y-TR1 cells treated with the HSP90 inhibitors 17-AAG and IPI-504 for 3 days (IC_50_=17-AAG, 650 nM (SH-SY5Y), 61 nM (SY5Y-TR1); IPI-504, 352 nM (SH-SY5Y), 35 nM (SY5Y-TR1)). (**b**) pRTK array analysis of SY5Y-TR1 cells treated with either DMSO or IPI-504 (1 μM) for 6 h. (**c**) Western blot analysis of AXL, total and pERK levels in SH-SY5Y and SY5Y-TR1 cells after treatment with 1 μM IPI-504 for the indicated times. (**d**) Western blot analysis of AXL and HSP90 expression levels in SH-SY5Y and SY5Y-TR1 cells treated with or without 1 μM IPI-504 for 6 h and from which AXL was coimmunoprecipitated with an anti-HSP90 antibody.

It has been shown that ALK inhibitor-resistant non-small-cell lung cancer cell lines are susceptible to heat-shock protein 90 (HSP90) inhibitors.^27^ Moreover, AXL is an acknowledged substrate of HSP90,^28^ prompting us to investigate the consequences of HSP90 inhibition in the context of NB cell resistance to TAE684. We therefore tested the effects of the HSP90 inhibitors geldanamycin (17-AAG) and its semisynthetic derivative, retaspimycin hydrochloride (IPI-504),^27^ on TAE684-resistant NB cells. SH-SY5Y-TR1 cells showed a tenfold increase in sensitivity to HSP90 inhibition (Figure 7a). To identify the HSP90 targets whose inhibition led to such a striking response in TAE684-resistant cells, we determined the phosphorylation status of several RTKs in SH-SY5Y-TR1 cells after treatment with IPI-504 (Figure 7b). Compared with DMSO-treated SH-SY5Y-TR1 cells, those treated with IPI-504 exhibited a loss of pAXL. EPHB2 phosphorylation was again seen to be increased in SH-SY5Y-TR1 cells (Figure 1c), but this effect was unchanged with IPI-504 treatment, suggesting that this RTK most likely was not involved in TAE684-mediated resistance.

The above results led us to ask whether the decreased phosphorylation of AXL upon HSP90 inhibition was coupled with degradation and loss of total AXL in SH-SY5Y-TR1 cells. Treatment with IPI-504 led to a time-dependent reduction of total AXL levels in SH-SY5Y-TR1 cells (Figure 7c). Importantly, the reduction in AXL levels on exposure to HSP90 inhibitor was accompanied by a concomitant decrease in pERK levels, again indicating that activated AXL signals at least partially through the MAPK pathway in TAE684-resistant cells (Figure 7c). HSP90 functions as a molecular chaperone that stabilizes AXL through direct binding.^28^ To establish whether the interaction between AXL and HSP90 in SH-SY5Y-TR1 cells was compromised by IPI-504, we coimmunoprecipitated HSP90 in resistant and parental cells before and after treatment with IPI-504 and analyzed AXL expression by western blotting with an anti-AXL antibody. Although no AXL protein bound to HSP90 in SH-SY5Y cells, binding to HSP90 was observed in SH-SY5Y-TR1 cells, and this was markedly decreased following treatment with IPI-504 (Figure 7d). Therefore, HSP90 inhibition in SH-SY5Y-TR1 cells leads to substantial reduction of AXL activity through diminished binding. Together, these results support the hypothesis that activated AXL mediates resistance to ALK inhibition in ALK^F1174L^-driven NB cells and suggests a therapeutic strategy to overcome such resistance.

## Discussion

Resistance to tyrosine kinase inhibitors of ALK has been described in multiple tumor types and can arise through different mechanisms.^1^ Here we show that in ALK^F1174L^-driven NB cells, the development of resistance to TAE684 and its clinically available derivative, LDK378, is associated with *AXL* overexpression and activation, as well as increased ERK signaling. The resistant cells exhibit increased sensitivity to an AXL inhibitor in comparison with TAE684-sensitive parental cells, with concomitant downregulation of ERK signaling. Aberrantly expressed AXL appears to be activated through increased levels of its ligand, GAS6, that also stabilizes the kinase. Resistance to TAE684 was associated with induction of an EMT phenotype. Finally, TAE684-resistant cells were significantly more sensitive to HSP90 inhibition, at least partly through its impact on AXL binding. Our findings not only identify a molecular mechanism of resistance to ALK inhibition in ALK^F1174L^-driven NB, but also suggest that effective AXL or HSP90 inhibitors, combined with a TAE684-derived ALK inhibitor, could provide a useful strategy to overcome this complication.

AXL is an RTK in the TAM kinase family whose members function as homeostatic regulators in adult tissues and play prominent roles in the nervous system.^15^ When AXL is activated by its ligand GAS6,^25^ it also contributes to key physiological processes such as cell survival, proliferation and migration^29^ mainly through the MAPK and PI3K signaling pathways.^30, 31^ *AXL* overexpression has been reported in various cancers and its role in regulating the actin cytoskeleton links this kinase to tumor invasiveness and metastasis, as shown in glioblastoma^32^ and breast cancer.^33, 34^ Moreover, *AXL* overexpression has been implicated in resistance to both standard and targeted anticancer agents (mainly epidermal growth factor receptor (EGFR) inhibitors) in various cancers, with or without accompanying EMT features.^16, 17, 35, 36, 37^

To our knowledge, this report provides the first evidence of AXL overexpression and activation as an acquired mechanism of resistance to ALK inhibition in NB. We would stress that the findings presented here are restricted to human NB cells in which the *ALK*^*F1174L*^mutation is the principal if not the sole driver of tumorigenesis. This model accounts for ∼2% of all NB cases at diagnosis^38^ but does not necessarily apply to cases in which *ALK*^*F1174L*^ coexists with other major genetic aberrations such as *MYCN* amplification. It will be important, therefore, to assess the significance of *AXL* overexpression and activation in cases of ALK-inhibitor resistance where the pathogenic role of *ALK* mutations is less dominant. Interestingly, in a study by Duijkers *et al.*,^39^ *AXL* overexpression was observed in established human NB cell lines that had not been exposed to targeted therapy, and its genetic depletion led to decreased cell migration and invasion, but not proliferation or downstream signaling.^39^ The decreased growth kinetics and downregulated pERK after AXL depletion in the TAE684-resistant cells likely reflect their relatively higher dependence on increased AXL activity.

Whether AXL inhibition alone is sufficient to reverse resistance to TKIs remains unclear. Genetic and pharmacological inhibition of AXL has been shown to restore sensitivity to erlotinib in *EGFR*-mutant lung cancer models with acquired erlotinib resistance, AXL activation and mesenchymal transition.^17^ However, in other studies, a functional role for AXL in both erlotinib-resistant *EGFR*-positive or crizotinib-resistant *EML4-ALK*-positive lung cancer cells with *AXL* overexpression and an EMT phenotype has been excluded.^40, 41^ Our results demonstrate that AXL inhibition by itself only partly rescues TAE684 resistance, although it resensitizes these cells to TAE684, similar to results seen in head and neck cancer cells with acquired erlotinib resistance.^35^ Thus, although AXL expression may contribute significantly to acquired resistance to TAE684, additional molecular mechanisms appear to be required for full development of the resistance phenotype.

Furthermore, the frequent association of *AXL* upregulation with an EMT phenotype in acquired TKI resistance^17, 35^ raises the intriguing question of whether AXL truly causes resistance or is merely a biomarker of EMT. In an analysis of multiple human cancer cell lines, in which elevated AXL was associated with a mesenchymal phenotype, EMT-associated drug resistance was found to be independent of AXL function.^41^ However, our results indicate that both the acquisition of EMT features and AXL activation are required to confer resistance to TAE684, although the relative contributions of these changes remain to be assessed. Overexpression of *TWIST2*, despite inducing an EMT phenotype, did not alter either AXL expression or sensitivity to TAE684 in parental SH-SY5Y cells, whereas overexpression of *AXL*, although not affecting TWIST2 expression levels, led to an EMT phenotype and modest resistance to TAE684. *TWIST2*, when combined with *AXL* overexpression, led to a greater decrease in TAE684 sensitivity compared with ectopic expression of *AXL* alone. These findings suggest that the activation of AXL is independent of TWIST2 upregulation in TAE684-resistant cells, but can act cooperatively to enhance the resistant phenotype in response to ALK inhibition. Of note, our results may be confounded by the fact that the gene expression patterns of ectopic overexpression of a single EMT-associated gene could differ from a phenotype that develops over time.

The mechanism of AXL activation appears to involve autocrine regulation through its ligand, GAS6. We observed increased levels of cleaved GAS6 protein in the supernatant of TAE684-resistant cells as compared with the parental cells, suggesting that the active form of the ligand is secreted in order to sustain AXL activation and stabilization. Similarly, in mesenchymal non-small-cell lung cancer cell lines with EMT features, resistance to erlotinib and upregulation of AXL was associated with markedly increased levels of GAS6.^16, 17^ Therefore, cleaved soluble GAS6 could be exploited as a biomarker of resistance to ALK inhibition. Indeed, if validated in human samples from ALK inhibitor-treated patients, the ability to specifically track cleaved GAS6 levels over time in the serum of patients would be a less invasive way to detect the early development of resistance.

We observed that TAE684-resistant cells were highly sensitive to HSP90 inhibition, partly due to depletion of AXL, leading to decreased downstream signaling. AXL was recently identified as an HSP90 substrate and was shown to be degraded in the intracellular compartment by geldanamycin.^42^ The increased vulnerability of these cells may reflect the presence of other proteins, including other RTKs that contribute to cell proliferation and survival, and by extension of the resistance phenotype, that are simultaneously disrupted with HSP90 inhibition. Our findings extend the currently emerging paradigm for the design of HSP90 inhibition-based strategies, either alone or in combination with selective ALK targeting, in the management of ALK-driven resistant cancers.

In ALK inhibitor-resistant cells, the ERK pathway appeared to be a major signaling mechanism through which activated AXL contributed to resistance. Whereas the PI3K/AKT/mTOR pathway is primarily involved in ALK downstream signaling in parental SH-SY5Y cells,^5^ pERK was upregulated in TAE684- and LDK378-resistant SH-SY5Y cells. Moreover, any alteration in AXL or GAS6 levels was closely correlated with changes in ERK signaling in the resistant cells: genetic and pharmacological depletion of AXL led to decreased ERK phosphorylation, as did shRNA knockdown of *GAS6*, and HSP90 inhibitor-induced depletion of AXL. Although activated AXL could utilize multiple downstream signaling pathways, our observations highlight the ERK pathway as an essential signaling node required to maintain cell survival in the face of targeted treatment. Indeed, two recent publications support the central role of ERK signaling in treatment resistance; first, MAPK pathway mutations are often frequently found in relapsed NBs after chemotherapy,^43^ and, second, several RTK ligands can confer resistance to kinase inhibitors (including TAE684) by reactivation of ERK in oncogene-addicted cancer cell lines.^44^ It is therefore reasonable to suggest that ERK inhibition with small molecules would be an effective strategy in therapy-resistant tumors or even to prevent the emergence of resistance through this mechanism.

Given the number of tumors that develop resistance to TKIs with upregulation of *AXL*, we propose that this mechanism is common to any tumor targeted with these agents, and that it should be considered in patients who develop such resistance. Especially in NB, where clinical trials of ALK inhibitors alone or in combination with standard chemotherapy agents are planned, it would be reasonable to assume that numerous instances of resistance will involve activation of AXL, and potentially the development of EMT. Indeed, AXL or GAS6 expression could be used as biomarkers of resistance in tumors that have not yet acquired the EMT phenotype. As EMT is driven by transcription factors that are currently undruggable, AXL activation represents an attractive target for inhibition in tumors resistant to ALK or other RTK inhibitors. As an alternative to an effective AXL inhibitor, depletion of this RTK could also be readily achieved by HSP90 inhibition.

## Materials and methods

### Cell lines and reagents

The human NB cell line SK-N-SH and its derivative SH-SY5Y were purchased from the American Type Culture Collection (Manassas, VA, USA) and their authenticity confirmed by genotyping. Cells were also confirmed to be mycoplasma negative. Parental and resistant cells were grown in RPMI media supplemented with 10% fetal bovine serum and 1% penicillin/streptomycin. TAE684 and R428 were synthesized in-house in Dr Nathanael Gray's laboratory (Boston, MA, USA). 17-AAG, CHX and LDK378 were purchased from Selleck Chemicals (Houston, TX, USA), and IPI-504 was purchased from APExBIO (Houston, TX, USA). Recombinant human GAS6 (rGAS6) was purchased from R&D Systems (885-GS-050; Minneapolis, MN, USA).

### Cell viability assay

Viability experiments were performed using the CellTiter-Glo Luminescent Cell Viability Assay (G7573; Promega, Madison, WI, USA) according to the manufacturer's instructions. All dose–response assays were performed in triplicate in 96-well plates and repeated at least three times. The results, representing the mean±s.d. of three separate biological experiments, were plotted as a nonlinear regression curve fit using Graphpad Prism 6 software (La Jolla, CA, USA). The x axis represents the log2 concentration of the indicated compound.

### Western blotting and immunoprecipitation

Cell lysates or conditioned media that were concentrated using Amicon Ultra 0.5 10K Centrifugal Filters (EMD Millipore, Billerica, MA, USA) were prepared using standard protocols. The following antibodies were used: AXL (4566), pAXL (5724), ALK (3333), pALK (3341), AKT (4691), pAKT (9271), ERK (4695), pERK (4377), VIM (5741), CDH1 (3195), tubulin (2128) and actin (4967) from Cell Signaling Technology (Danvers, MA, USA); GAS6 (sc-376087), HSP90 (sc-59577) and phosphotyrosine (sc-81529) from Santa Cruz Biotechnology (Santa Cruz, CA, USA); Flag (F3165) from Sigma-Aldrich (Saint Louis, MO, USA); and AXL antibody for immunocytochemistry from R&D Systems (AF154).

### Phospho-RTK array analysis

Cell lysate (500 μg) was incubated on a human phospho-RTK membrane array (ARY001B; R&D Systems) according to the manufacturer's instructions. Target proteins were captured with their respective antibodies. After washing, the proteins were incubated with a phosphotyrosine antibody conjugated to horseradish peroxidase to allow the detection of captured phospho-RTKs.

### Invasion assay

A cell suspension containing 5 × 10^5^ cells/ml in serum-free medium was added to the upper chamber of the invasion assembly (ECM550; Chemicon International, Billerica, MA, USA) and 10% fetal bovine serum containing media added to the lower chamber to act as a chemoattractant. After incubation for 48 h, non-migrating cells in the upper chamber were removed with cotton swabs, and cells that migrated to the lower surface of the filters were stained with crystal violet. The results were quantified by counting and averaging three independent fields per condition, and represent the mean±s.d. of three separate biological experiments.

### Lentiviral/retroviral transduction and transient transfection

Lentiviral-based pLKO.1 shRNA constructs (*shAXL*, *shERK* and *shGAS*6) were obtained from the RNAi Consortium of the Broad Institute (Cambridge, MA, USA). The constructs were transfected into 293T cells with helper plasmids for virus production. Cells were then transduced with virus, followed by puromycin selection for at least 3 days. Stable overexpression of the retroviral-based pMSCV vector containing *TWIST2*, *AXL* or both genes was performed similarly. Transient knockdown of *AXL* was performed with specific Silencer Select siRNA (Life Technologies, Carlsbad, CA, USA) according to the manufacturer's instructions. GAS6 siRNA pool was purchased from Dharmacon RNAi Technologies (Lafayette, CO, USA).

### Gene expression analysis

Three biological replicates of total RNA were isolated from SH-SY5Y and SY5Y-TR1 cells using the RNeasy Mini kit (Qiagen, Valencia, CA, USA). Total RNA was hybridized to GeneChip Human Genome U133 Plus 2.0 Arrays (Affymetrix, Santa Clara, CA, USA), according to the manufacturer's instructions. The data obtained are accessible through the GEO accession number GSE73292. Data analysis was performed using GenePattern software.^45^ GSEA was performed with the GSEA application,^46^ using log2 fold change to rank genes.

### Immunocytochemistry

1 × 10^6^ cells were formalin fixed and immunocytochemistry performed based on established protocols.^9^ AXL antibody (R&D Systems) in a 1:1000 dilution was used to determine AXL expression.

### Quantitative RT–PCR

Total RNA was isolated using the RNeasy Mini kit (Qiagen), followed by RT–PCR with the ThermoScript RT–PCR system (Life Technologies). Quantitative PCR was carried out using the QuantiFast SYBR Green PCR kit (Qiagen) in a 96-well plate format, and analyzed on an Applied Biosystems ViiA 7 Real-Time PCR System (Life Technologies). Each sample was run in triplicate and normalized to actin as an internal control. Relative quantification was calculated according to the ΔΔCt relative quantification method. The results represent the mean±s.d. of three separate biological experiments. Primer sequences are available upon request.

### Sequence analysis

The kinase domain of ALK and full-length AXL were amplified from complementary DNA of SH-SY5Y and SY5Y-TR1 cells using the HotStar HiFidelity Polymerase Kit (Qiagen). The PCR products were cloned into the pGEM-T vector (Promega) and confirmed by sequencing. The GAS6 insertion (GAS6-SV) was similarly amplified from the two cell lines, and the gel-purified PCR product confirmed by sequencing.

### Statistical analysis

Statistical significance for all comparisons between two groups was determined with the two-sided Student's *t*-test: *P<0.05, **P<0.01 and ***P<0.001. The effect of combining TAE684 and R428 was determined using the Bliss additivity model.^47^
