# Supplementary material for: ALK inhibitor resistance in ALKF1174L-driven neuroblastoma is associated with AXL activation and induction of EMT
Source: Oncogene. 2015 Nov 30;35(28):3681–91. doi: 10.1038/onc.2015.434 (PMC4885798; doi:10.1038/onc.2015.434)
Supplement: Supplementary Figure Legends [file onc2015434x1.pdf]

Supplementary Figures

Supplementary Figure S1. Development of TAE684-resistance in ALK-mutated SH-SY5Y NB cells. (**a**) Dose-response curves for parental SH-SY5Y and SY5Y-TR cells (mixed population), maintained in 250 nM of TAE684 or in drug-free media for 10 passages (SY5Y-TR-ND), treated with increasing concentrations of TAE684 for 3 days. The results represent the mean ± SD of three separate experiments. (**b**) Immunoblot analysis of total and pALK and its indicated downstream signaling molecules in parental SH-SY5Y and TAE684-resistant SY5Y-TR cells.

Supplementary Figure S2. Development of TAE684-resistance in ALK-mutated SK-N-SH cells. (**a**) Dose-response curves for parental and TAE684-resistant SK-N-SH (SK-N-SH-TAE-R) cells treated with TAE684 for 3 days [IC_{50} = SK-N-SH, 35 nM; SK-N-SH TAE, 102 nM]. (**b**) Western blot analysis of total and pALK, AXL and ERK proteins in TAE684-sensitive and resistant SK-N-SH cells. (**c**) Dose-response curves for parental and SK-N-SH-TAE-R cells treated with R428 for 3 days [IC_{50} = SK-N-SH, 865 nM; SK-N-SH TAE, 448 nM].

Supplementary Figure S3. Analysis of AXL activation in TAE684-resistant SY5Y-TR1 cells. (**a**) Quantitative PCR analysis of genomic *AXL* levels in SH-SY5Y and SY5Y-TR1 cells. DNA was isolated from cells using QIAamp DNA mini kit (Qiagen) and qPCR performed as described in the Materials and Methods. (**b**) qRT-PCR analysis of the indicated miRNAs in SH-SY5Y and SY5Y-TR1 cells. Total RNA was extracted using Trizol and the miScript PCR system kit (Qiagen) was used for qRT-PCR. The results are expressed as mean ± SD of three separate experiments ***P<0.001. (**c**) qRT-PCR analysis of miRNA-199b and AXL, following the exogenous expression of miR-199b mimic (Sigma Aldrich) in resistant SY5Y-TR1 cells. Hsa-miR-199b mimics (50 nM) were transfected as described in the Materials and Methods.

The results are expressed as mean ± SD of three separate experiments ****P*<0.001. (**d**) Western blot analysis of AXL and total and pERK levels in TAE684-resistant cells in which miRNA-199b was overexpressed at increasing doses and for varying times.

Supplementary Figure S4. GAS6 upregulation leads to AXL protein stabilization. (a) qRT-PCR analysis of AXL expression levels in parental SH-SY5Y cells overexpressing AXL, and treated with three different concentrations (100, 200, 400ng/mL) of recombinant human GAS6. Untreated (U) SH-SY5Y cells were used as a reference. The results are expressed as mean ± SD of three separate experiments. ***P<0.001. (b) Immunoblot analysis of total AXL levels in AXL-overexpressing SH-SY5Y cells pretreated with rGAS6 (400ng/mL) for 3 days, and then exposed to 200uM of the protein synthesis inhibitor cycloheximide (CHX) for the indicated times.
