# Supplementary figures and images for: ALK inhibitor resistance in ALKF1174L-driven neuroblastoma is associated with AXL activation and induction of EMT
Source: Oncogene. 2015 Nov 30;35(28):3681–91. doi: 10.1038/onc.2015.434 (PMC4885798; doi:10.1038/onc.2015.434)

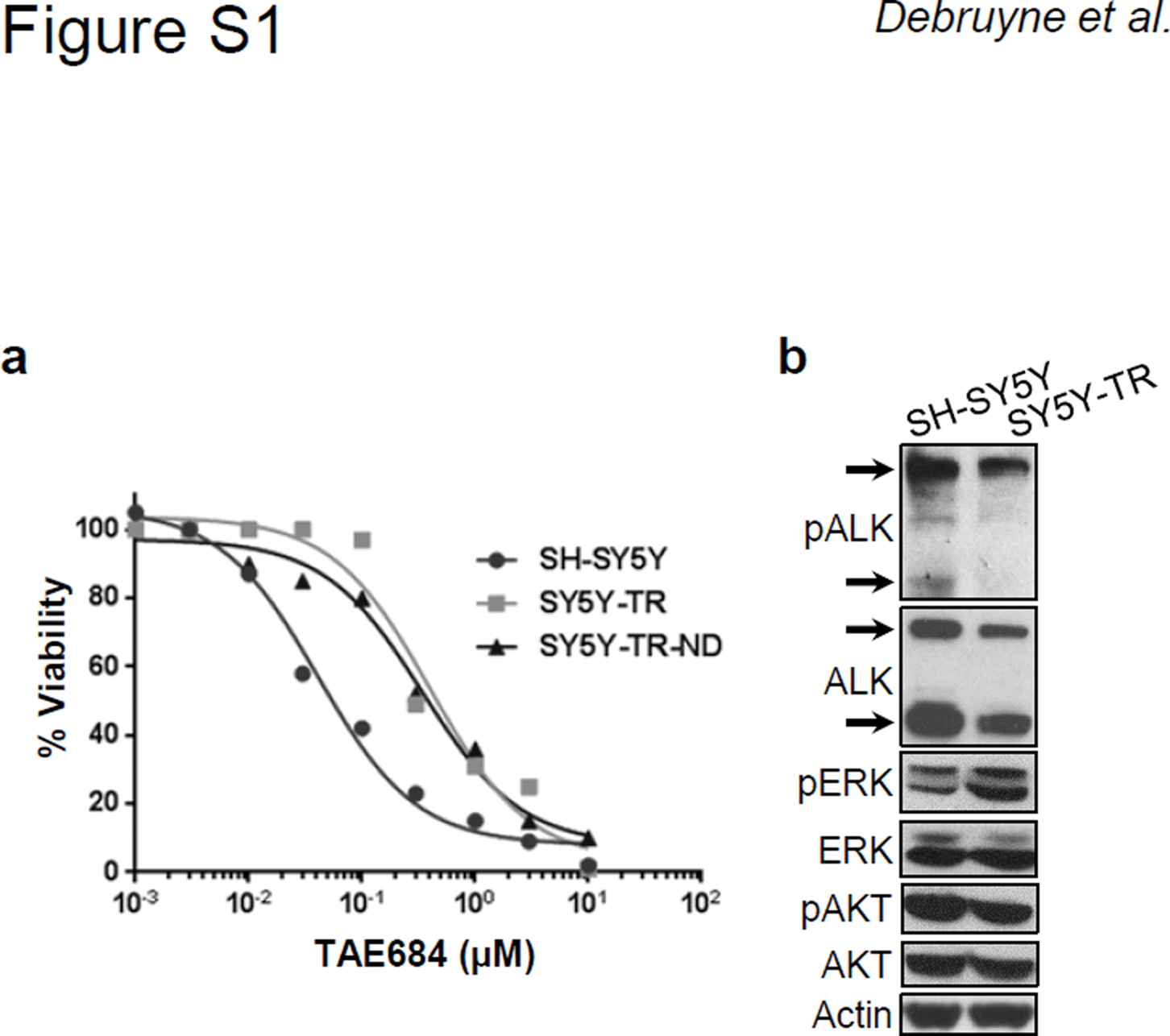

Supplement: Supplementary Figure S1 [file onc2015434x2.tif]

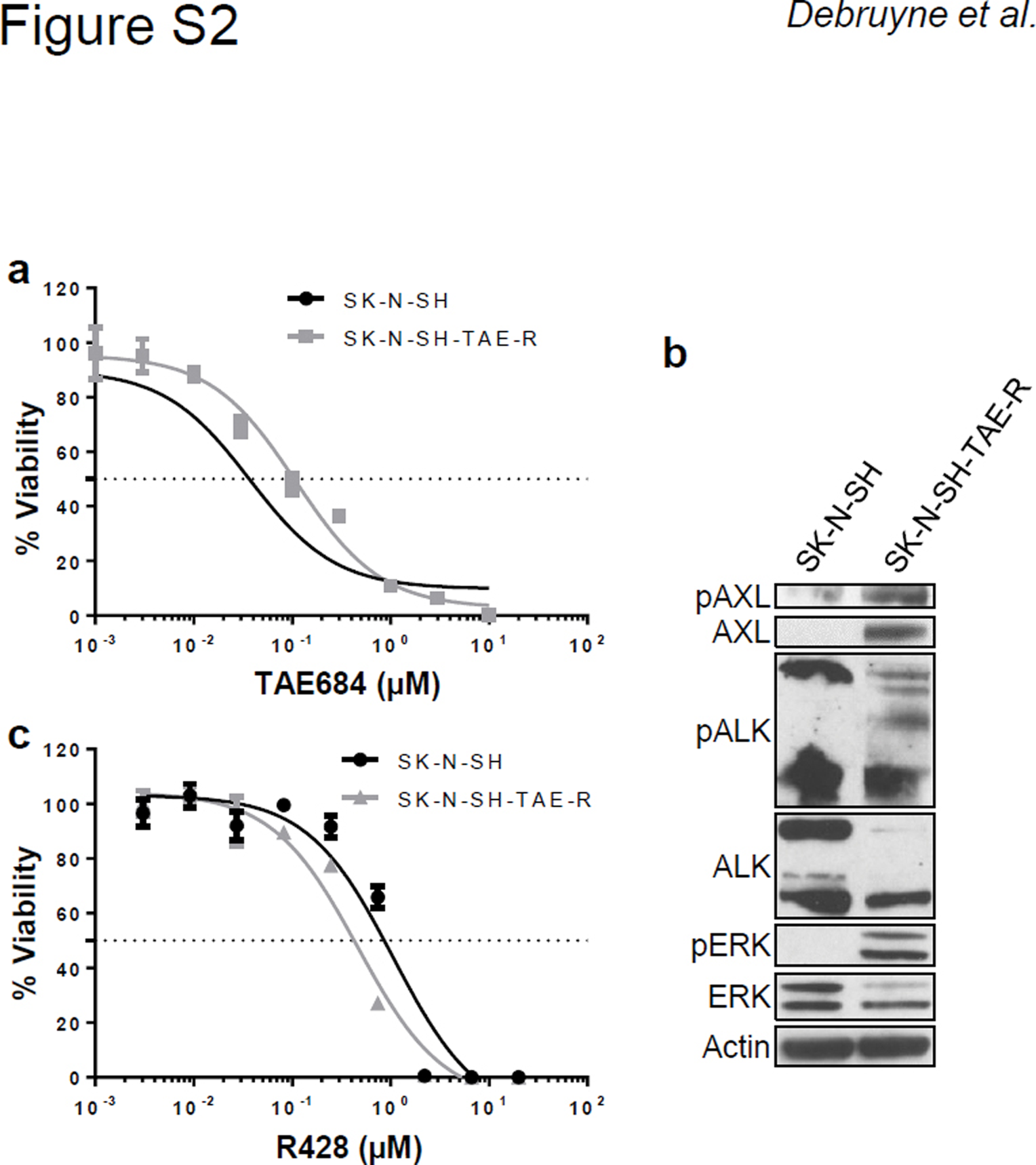

Supplement: Supplementary Figure S2 [file onc2015434x3.tif]

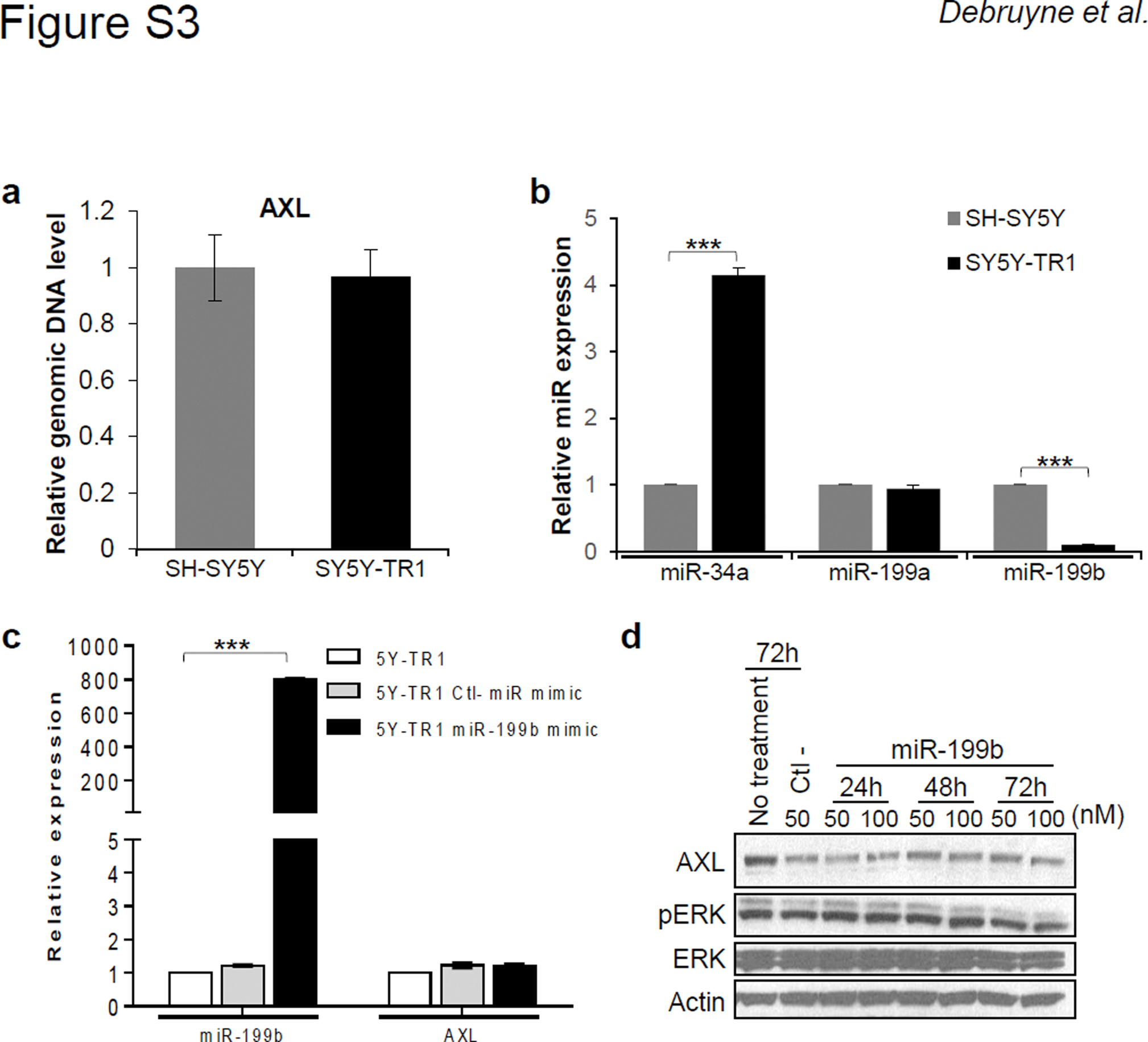

Supplement: Supplementary Figure S3 [file onc2015434x4.tif]

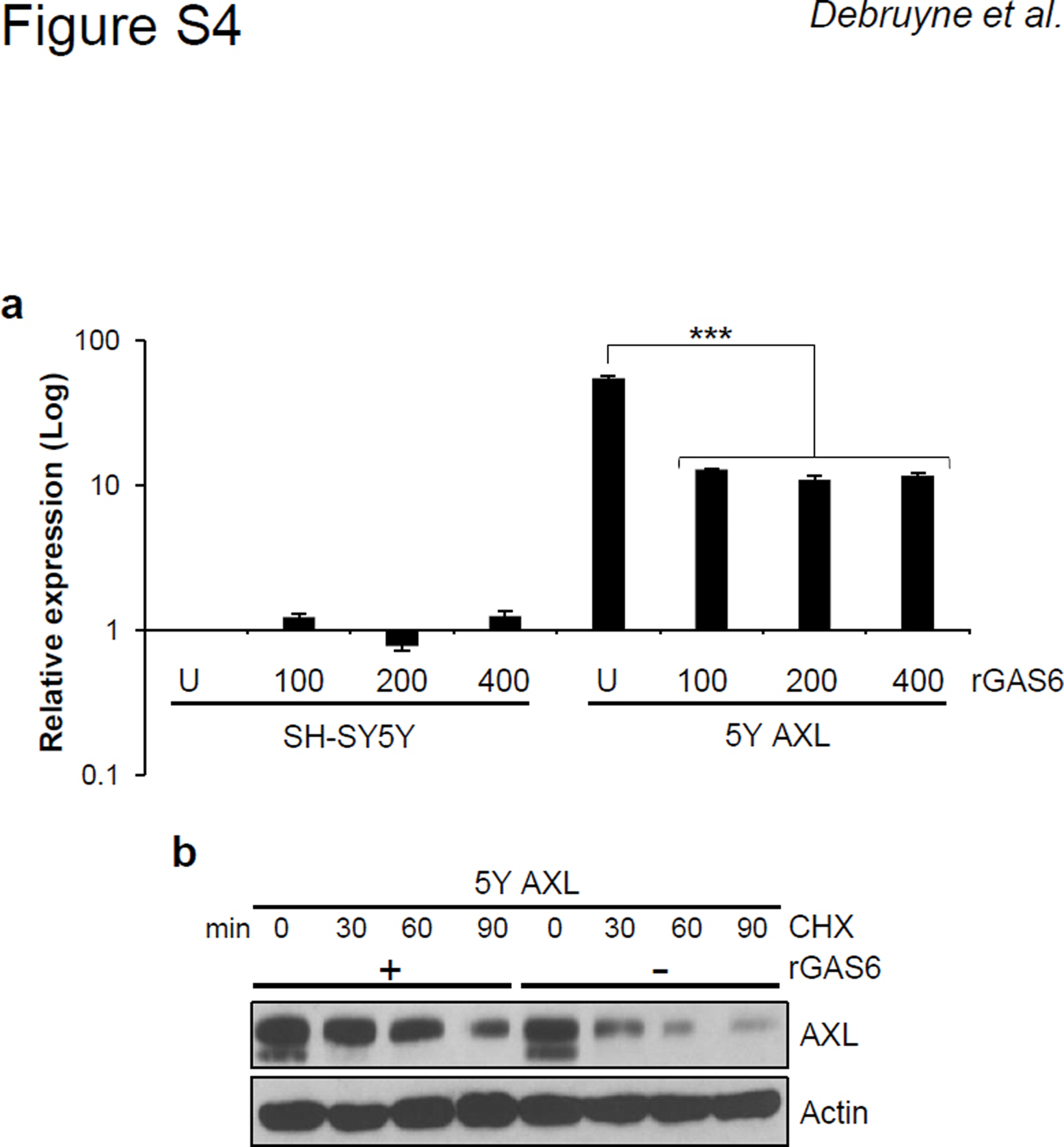

Supplement: Supplementary Figure S4 [file onc2015434x5.tif]
